# Parental Pressure, Family Support and Household Workload Among High School Students in Rural Parts of Nepal: An Observational Study

**DOI:** 10.31729/jnma.v63i292.9245

**Published:** 2025-12-31

**Authors:** Bipsana Shrestha, Minani Gurung

**Affiliations:** 1 Institute of Medicine, Tribhuvan University, Kathmandu, Nepal; 2 Kathmandu University School of Medical Sciences, Dhulikhel, Kathmandu, Nepal

**Keywords:** *academic performance*, *family support*, *high school students*, *household workload*, *parental pressure*

## Abstract

**Introduction:**

When parents have higher academic-related demands from their children and when children have higher household workload, academic stress occurs. Consequently, student cannot concentrate on their studies and hamper their academic performance. Nonetheless, family support can be a vital contributor to academic success of a child. Therefore, the objective of this study was to assess the level of parental pressure, family support and household workload in high school students in rural part of Nepal.

**Methods:**

A cross-sectional study was conducted in Thabang village of Rolpa district among 521 students of class 11 and 12 using a self-structured questionnaire based on literature reviews. The questionnaire for parental pressure had 10 items, for household workload had 6 items and for family support had 7 items. The data collection period was from June to August 2019. Ethical approval was taken from Nepal health research council (reference no. 233/2019). Descriptive statistics were reported to find out the frequency, percentage, mean, and standard deviation.

**Results:**

Findings of this study showed that there was low parental pressure among 242 (46.45%) students, low family support among 240 (46.07%) students, and high household workload among 135 (25.91%) students.

**Conclusions:**

The participants had low parental pressure in academic areas as well as low family support but they had high workload of household chores. High household workload and low family support may lead to academic stress in students which can hamper academic performances of the students.

## INTRODUCTION

When parents have higher academic-related demands from their children than the actual capabilities of the students, there occurs an academic stress.^1^ Studies from India suggest that although parents’ involvement affects the academic outcomes positively,^2^ excessive expectations can lead to anxiety in students as they directly or indirectly feel pressurized to achieve academic success.^1^ Consequently, it becomes difficult for the students to concentrate on regular studies and resulting stress, anxiety can even lower their academic performance.^3^ Nonetheless, support from parents or guardians can be a vital contributor to academic success of a child.^4,5^ Further, another area to explore regarding academic outcomes is the household workload among students. Study from Pakistan reported that students performing regular household chores are more likely to get poor academic scores.^6^ Therefore, the objective of this study was to assess the level of parental pressure, family support and household workload in high school students in rural part of Nepal.

## METHODS

A cross sectional study was conducted to assess the prevalence of parental pressure, family support and household workload among high school students. It was done in Thawang village of Rolpa district. Thawang rural municipality of Lumbini Province of Nepal was established in 2016/17 AD. It has an area of 191.07 square kilometers and has 2399 households. The total population of the municipality was 10,881 according to census of Nepal in 2011. It is one of the remote rural municipalities of the Lumbini Province of Nepal.^7^ Students of class 11 and 12 were taken as the study population. Those having mental health problems and/or could not communicate verbally were set as the exclusion criteria. There a total of 22 high schools in Thawang village, out of which six were selected conveniently. All students of class 11 and 12 from six high schools of the village participated in this study, making a total sample size of 521. Prior to the collection of data, there was an orientation of six teachers which covered the objectives of the research, and data collection procedures. Additionally, a parent teacher meeting was also conducted to explain about the study and its process. At the end of the meeting, consent for their children to participate in the study was taken. Assent from the students were also taken. Structured questionnaire was distributed to the students in the class. The data collection was done on a school day during the last period. The teachers of the school assisted in answering any queries and misunderstandings in the questionnaires. Informed consent and voluntarism were considered at the top during data collection. The right to participation or reject along with confidentiality was strictly followed. The respondent spent approximately 20-30 minutes with the questionnaires. A self-structured questionnaire was used which was based on the conceptual framework and literature reviews, and they were modified according to the context of the research site and background. The questionnaire for parental pressure had 10 items and it had four options ‘strongly agree’ scored ‘4’, ‘agree’ scored ‘3’, ‘disagree’ scored ‘2’ and ‘strongly disagree’ scored ‘1’. The score for parental pressure ranged from 1-40.

Scores were categorized as below following Bloom’s criteria^8^; High parental pressure: ≥ 80% ( ≥ 32 score)

Moderate parental pressure: 60-79% (24-31 scores)

Low parental pressure: ≤ 59% (≤23 scores)

The questionnaire for household workload had 6 items and it had three options for the respondents: ‘always’ ‘occasionally’ and ‘never’. Since all the statements were negative the scoring system for the response ‘always’ was scored 2, ‘occasionally’ was scored 1 and ‘never’ was scored 0. The score ranged from 0-12.

Scores were categorized as below following Bloom’s criteria;

High workload: ≥80% (≥11)

Moderate workload: 60-79% (8-10)

Minimum workload: ≤ 59% (≤7)

The questionnaires for family support had 7 items and the respondent had four options ‘strongly agree’ ‘agree’ ‘disagree’ and ‘strongly disagree’. Scoring system for the response ‘strongly agree’ is 4 which is the highest score, for ‘agree’ is 3, ‘disagree’ is 2 and ‘strongly disagree’ is 1 which is the lowest score as it represents strong negative response.

Scores were categorized as below following Bloom’s criteria;

Good family relations: ≥ 80% (≥ 26 scores)

Moderate family relations: 60-79% (19-25)

Low family relations: ≤ 59% (≤ 18 scores)

For reliability, data was pretested on 10% of the sample size in Nepal Police school, Sanga, a site in outskirt of Kathmandu valley that reflected the population of Rolpa. The comments and response from the pre-test were used to modify and adjust the questionnaire. The study duration was from June to October 2019, and the data collection period was from June to August 2019. Ethical approval was taken from Nepal health research council (reference no. 233/2019). Data analysis was done with SPSS 18 software. Before the data entry, raw data was checked for consistency and missing information. After that data coding and verification was done. Descriptive statistics were reported to find out the frequency, percentage, mean, and standard deviation.

## RESULTS

The age of the participants ranged from 17 years to 19 years. The mean age was 18.38 years with the standard deviation of 0.78. There were 294 (56.43%) participants who were of age 19 years, 131 (25.14%) who were of age 18 years, and 96 (18.43%) who were of age 17 years. There were 352 (67.56%) male students and 169 (32.44%) female students. Among all the participants, 205 (39.35%) were from class 11 and 316 (60.65%) were from class 12. Regarding the type of family of the students, 325 (62.38%) belonged to an extended family and 196 (37.62%) belonged to a nuclear family (Table 1).

**Table 1:** Sociodemographic characteristics (n= 521).

Characterictics	n(%)
**Age (in years)**	
17	96(18.43)
18	131(25.14)
19	294(56.43)
**Sex**	
Male	352(67.56)
Female	169(32.44)
**Class**	
11	205(39.35)
12	316(60.65)
**Family type**	
Extended	325(62.38)
Nuclear	196(37.62)

Table 2 shows that the low parental pressure was reported by 242(46.45%) students, moderate pressure was reported by 128 (24.57%)students, and high pressure was reported by 151 (28.98%) students.

**Table 2:** Level of parental pressure in the participants (n=521).

Parental Pressure	n(%)
Low pressure (≤23)	242(46.45%)
Moderate pressure (24-31)	128(24.57%)
High pressure (≥32)	151(28.98%)

Table 3 shows that the low family support was reported by 240 (46.07%) students, moderate supportwasreportedby 126 (24.18%) students, and high support was reported by 155 (29.75%) students.

**Table 3:** Level of family support in the participants (n=521).

Family Support	n(%)
Low support (≤18)	240(46.07%)
Moderate support (19-31)	126(24.18%)
Good support (≥32)	155(29.75%)

Table 4 depicts that there was a presence of high household workload in 135 (25.91%) participants. Moderate workload was present in 81 (15.55%) participants, and there was minimum household workload in 305 (58.54%) participants.

**Table 4:** Level of household workload in the participants (n=521)

Household Workload	n(%)
Minimum workload (≤7)	305(58.54%)
Moderate workload (8-10)	81(15.55%)
High workload (≥11)	135(25.91%)

## DISCUSSION

The findings of this study showed that in most of the participants (46.45%), there is low parental pressure while high parental pressure was found in 28.98%. On the contrary, a study conducted among high school students of Jagobiao National High School in Philippines showed that the students had high level of parental pressure such that parents expected higher academic achievements from their children. It also mentioned that extreme pressure from the parents can lead to stressful situations in a student’s life.^9^ This can ultimately lead to depression, anxiety, lower self-esteem, and emotional withdrawal like mental health issues in students.^10,11^ Sometimes, it can even lead to suicidality like highly unexpected and undesirable outcome.^12^ Therefore, firstly, every parent must calibrate their child’s capacity before setting higher expectations of academic performance from the child.^9^ Substantially low parental pressure in our study might be because of the fact that it was conducted in one of the most rural parts of Nepal. Hence, the parents are more concerned about who could support their family in household and field work to earn enough money in order to run their daily lives, and less concerned about the academic achievements of their children.

Further, the findings of this study revealed low family support in 46.07% of the students regarding their study. However, this contradicts with the findings from the Gingoog City division of Philippines, where overall family support level was high for the students. The support from family included academic, mental, emotional and financial support which were provided all the time.^13^ This difference in familial support between Nepal and Philippines might be because of the difference in educational level of parents, which is even more prominent when compared to developing countries^14^ and highly educated parents expect highly of their children regarding academic success.^15^ They are able to provide more stimulating environment for their children to learn as they are more sensitive to their children’s academic needs. That is why highly educated parents spend more time with their children which provides assistance for the students.^15^ The study of Gingoog City division suggests a significant relationship between academic performance of the students and the family support they receive.^13^ Therefore, in developing countries like Nepal, where parents in rural parts are not well educated, it is essential to improve parental support for high school students. It is because education is an integral part of adolescents’ lifelong development which guides success in future academic path and careers of a person.^4,16^

Moreover, our findings showed that 25.91% of students had high household workload and 58.54% of them had minimum household workload. This was found to be comparable with the findings of a study done in Pakistan where 27.4% of the students reported that they often performed household chores. Further, it revealed that 12% of the students always experienced stress because of the household chores, and 18.2% of the students were even involved in conflict at their home due to the household chores.^17^ This stress might be because students who have higher workload at home do not get enough time either to complete their homework, or to revise their lessons and prepare for examinations. This in turn negatively affects the academic performance of students in long term. A study done in Portugal showed that working both at home or in the labor market is detrimental to a child’s academic performance, as measured by their test scores in Portugese and Mathematics.^18^ Some of the strengths of this study are that all the students of six high schools were included in this study, and pretesting was done in 10% of the sample in a comparable school which helped in refining and contextually adjusting the questionnaire. Despite these strengths, there are also few limitations of this study. Since this study was conducted in a single village of a rural part of Nepal, the results cannot be generalized to other regions or to population residing in urban area who might differ in socioeconomic and cultural aspects. Similarly, since the questionnaire was self-administered to the students, the results might have been influenced by the social desirability bias

## CONCLUSION

Although, the students in rural areas mostly have low parental pressure in academic areas, they also have low family support. In addition, just more than a quarter of the students also had high workload of household chores. The reason behind this might be the lower educational level of parents in rural areas of Nepal which hinders them from understanding the effects of parental pressure, family support and household workload on academic achievements of their children. Therefore, more studies should be conducted in future to explore these factors in students in rural as well as urban areas which will help in finding out the ways to support academic performances of needy students. Moreover, higher household workload and lower family support may lead to academic stress in students which can escalate to the more severe situations such as suicidality. Therefore, appropriate interventions such as counseling, talk sessions and group discussions must be administered in order to help high school students cope with academic stress, as well as provide support in order to improve their academic performance.

## Data Availability

The data are available from the corresponding author upon reasonable request.
